# *A Purchaser’s Guide to Clinical Preventive Services:* A Tool to Improve Health Care Coverage for Prevention

**Published:** 2008-03-15

**Authors:** Dyann M Matson Koffman, Andrew Lanza, Kathryn Phillips Campbell

**Affiliations:** Public Health Educator/Scientist, Division for Heart Disease and Stroke Prevention, National Center for Chronic Disease Prevention and Health Promotion, Centers for Disease Control and Prevention; McKing Consulting Corporation and Business Sector Partnerships and Strategic Alliances, the National Center for Health Marketing, Centers for Disease Control and Prevention, Atlanta, Georgia; National Business Group on Health, Center for Prevention and Health Services, Washington, District of Columbia

## Abstract

**Introduction:**

In 2005, representatives from the Centers for Disease Control and Prevention partnered with the National Business Group on Health and the Agency for Healthcare Research and Quality to form a work group for developing *A Purchaser's Guide to Clinical Preventive Services: Moving Science into Coverage.* This guide, designed as a tool for employers, describes recommended clinical preventive services for 46 conditions. The guide includes the scientific evidence and benefits language that employers need to include comprehensive clinical preventive services in their medical benefit plans.

**Methods:**

The work group determined that the guide would address conditions that 1) affected a large percentage of the working population, 2) were costly to control, and 3) had well-defined and accepted recommendations for preventive services. Subject matter experts from the Centers for Disease Control and Prevention, the National Business Group on Health, and the Agency for Healthcare Research and Quality developed or reviewed statements of scientific evidence for 46 diseases and conditions.

**Results:**

The *Purchaser's Guide,* written for an employer audience, includes descriptions for recommended clinical preventive services and their cost savings, syntheses of supporting evidence, strategies for prioritization, and recommendations to improve the delivery and use of preventive services. Twelve hundred copies were sent to more than 275 members of the National Business Group on Health and other purchasers of health care; training sessions on the *Guide* were held for 228 business leaders, health benefit consultants, and health plan administrators; and an online version was created through the Web sites of the National Business Group on Health and the Centers for Disease Control and Prevention. The online version has received more than 260,000 hits since its release.

**Conclusion:**

In 2007, the National Business Group on Health reported that some Fortune 500 companies will be using the *Purchaser's Guide* when negotiating their health benefit contracts and developing their health care strategies. Further research is under way to determine whether the *Guide* influences employers to purchase recommended clinical preventive services.

## Introduction

Researchers estimate that 75% of all health care costs stem from preventable chronic health conditions, yet only 1% of the money spent on health care in the United States is devoted to protecting health and preventing illness and injury (1-3). In addition, chronic health conditions result in substantial indirect costs for employers, such as lost productivity, absenteeism, and turnover (1). As Americans' health care expenditures continue to increase, strategies that reduce the prevalence and cost of preventable diseases become important. Prevention, early detection, and management of diseases can improve our nation's health and reduce health care costs (3).

Clinical preventive services, such as screenings, and counseling and treatment for risk factors such as hypertension, lipid disorders, and tobacco use, can help people avoid disease and reduce their health risks. Yet health care insurance benefits have historically been designed to address the treatment of diseases, not their prevention (4). National studies have found that adults in the United States receive approximately 55% of the recommended preventive care (4,5).

A 1997 survey of 3156 employers revealed that most employer-sponsored health plans did not cover many of the recommended clinical preventive services (6). In a more recent study that used 2001 data from 2180 employers, coverage of physical examinations, immunizations, and screenings generally exceeded 50%. Coverage of services that involved lifestyle modification (e.g., nutrition counseling) was less than 20%, however, even though employers understood the relationship between increased productivity, decreased health care costs, and coverage for clinical preventive services. In reality, employers in this study were least likely to cover the services that were most likely to result in a return on their investment (7). Another concern is that employers who do cover preventive services often use vague contract language (7-8).

Coverage for preventive care can determine whether people receive preventive services (9,10). For example, a national study of more than 1600 women found that women without health insurance had a worse risk factor profile for cardiovascular disease and used health care services less frequently than did those with health insurance (11). Another study of U.S. workers found that higher-wage workers were more likely than lower-wage workers to have health insurance and health-related benefits and to use preventive services (12). The absence of coverage can also have a serious effect on whether physicians offer clinical preventive services. In one study, 31% of physicians reported that they sometimes did not offer services to their patients because of perceived coverage restrictions (13).

Conversely, improvements in insurance coverage may increase access to, and use of, preventive health services (9,14-16). For example, several years after Medicare began covering preventive services, older adults without supplemental coverage were approximately 10% less likely to use preventive services such as cholesterol testing than those who had supplemental coverage (17). In terms of behavioral health, managed health plans increasingly include coverage for prevention and health promotion services (18). Even though employers understand the need to prevent illness and disability, they rarely cover important preventive benefits, and they may not follow evidence-based recommendations (7). Faced with rising health care costs, an aging workforce, and global competition, employers are more reliant on retaining employees in the workplace past retirement age and on keeping all their employees healthy, productive, and engaged (6,19).

In response to these issues, in 2006 the Centers for Disease Control and Prevention (CDC) and the National Business Group on Health (NBGH), in collaboration with the Agency for Healthcare Research and Quality (AHRQ), developed *A Purchaser's Guide to Clinical Preventive Services: Moving Science into Coverage* (19). This document is a comprehensive tool for employers, particularly those who make decisions about which health benefits to purchase. It provides guidance for the selection of clinical preventive services and ways to manage disease risk that the U.S. Preventive Services Task Force (USPSTF), CDC, and other authoritative organizations have determined are effective. The guide includes the scientific evidence and benefits language that employers need to include comprehensive clinical preventive services in their medical benefit plans.

The purpose of this article is to give an overview of the *Purchaser's Guide* and how to use it. We use a scenario that illustrates how an employer with several thousand employees might use the guide, and we highlight the recommended clinical preventive services and strategies that promote their delivery and use.

## Methods

In February 2005, representatives from CDC, in partnership with NBGH, discussed the feasibility of developing a decision support tool to assist managers of employee benefits and human resources in selecting and defining the appropriate benefits for clinical preventive services for their employees, dependents, and retirees. Through its established relationship with the NBGH, CDC learned that even NBGH members that were sophisticated and global Fortune 500 companies lacked the information to select, define, and implement evidence-based preventive services as benefits. NBGH members requested information about clinical preventive services that would improve their employees' health and ultimately reduce associated health care costs. At the same time, an emerging movement toward consumer-directed health plans and health savings accounts (HSAs) has required employers to specify the clinical preventive services they will cover at no cost to their beneficiaries; this practice is known as "safe-harbor" coverage. For these reasons, we decided to develop a document to guide businesses in the selection of clinical preventive services.

Representatives from the CDC and NBGH formed a work group to develop the *Purchaser's Guide*. Members determined that the guide would address conditions that 1) affected a large percentage of the working population, 2) were costly to control, and 3) had a well-defined and accepted recommendation for preventive service. In the summer of 2005, the work group made three important tactical decisions. First, it included AHRQ to provide guidance and direction. Second, it expanded the inclusion criteria to all USPSTF A and B recommendations (20). Third, the group decided on a standard format for each chapter, which significantly improved the readability and consistency of information in the *Purchaser's Guide.*

Beginning in the summer of 2005 and going through the early fall of 2006, the work group appointed subject matter experts, NBGH staff, or both to write evidence statements for 46 health conditions and for other chapters. The writers also developed specific benefit language, known as summary plan descriptions (SPDs). Employers and managers in employee benefits or human resources need SPDs to ensure that they include a comprehensive clinical preventive services program in their medical benefit plan. Work group members extensively reviewed each chapter, submitted the evidence statements through the CDC clearance process, and delivered the text to NBGH for final printing. In August 2006, approximately 12 employers, consultants, and experts in prevention reviewed a draft of the guide; their feedback was used in making revisions before the guide was published.

## Results

At a press conference held on November 28, 2006, the executive leadership of NBGH, CDC, and AHRQ released the *Purchaser's Guide and *sent 1200 copies to more than 275 NBGH members and other purchasers of health care. Three "webinars" (seminars on the Web), training sessions, and presentations for 228 NBGH business leaders, health benefits consultants, and health plan administrators were conducted. The *Purchaser's Guide* is publicly available online through the NBGH (www.businessgrouphealth.org/prevention/purchasers) and CDC Web sites (www.cdc.gov/business) and has received more than 260,000 hits since its release. In 2006, the University of Washington's Health Promotion Research Center was funded to conduct a formal evaluation of the *Purchaser's Guide* in the areas of employers' purchasing decisions and coverage for preventive services.

Chapter 1, "The Role of Clinical Preventive Services in Disease Prevention and Early Detection," provides the rationale for endorsing clinical preventive services and an overview of all seven chapters in the *Purchaser's Guide* (Table 1). Because many employers will not be able to cover all 46 clinical preventive services as benefits, we present some ways to prioritize services, as recommended in Chapter 4, "The Prioritization and Strategic Implementation of Clinical Preventive Service Benefits." Employers can use each of these methods or a combination of them to determine which clinical preventive services would be most helpful to their beneficiaries.

**1) Determine which clinical preventive services have the greatest economic and health value.** The top 20 "high-value" preventive services, rank-ordered according to their cost-effectiveness and ability to prevent disease, injury, or premature death, are shown in Table 2 (5). Some high-value services, such as vision screening for adults older than 65 years, are not included in the *Purchaser's Guide* because only a small proportion of covered employees fall into that age group.

**2) Address the workforce's demographic characteristics and health conditions.** Employers can evaluate whether their current clinical preventive benefits meet the needs of their beneficiary population. Employers with heterogeneous populations may decide to choose among the top 20 high-value preventive services. Employers with populations that are demographically homogeneous (e.g., mostly male or mostly female, mostly young adults [aged 20 to 40 years], or mostly older adults [aged 50 to 70 years]) may decide to tailor their choice of clinical preventive services benefits to their particular population. For a brief summary of clinical preventive services appropriate for different categories defined by age group and sex, employers can refer to the charts in chapter 7, "Resources and Tools."

**Box 1. Sample Scenario**

Employer A has 4000 employees, 6000 dependents, and 1000 retirees. The employer provides coverage for all three groups, including Medicare Part B for retirees. The beneficiary population represents diverse racial/ethnic groups; 49% are female, and the average age is 38 years. After a review of the health risk appraisal (HRA) information and medical claims data, several diseases and conditions emerge as major health or cost problems. Heart attacks, strokes, diabetes, and depression account for a high proportion of health care claims and a substantial percentage of absenteeism-related costs. The HRA data reveals that the prevalence of hypertension and lipid disorders is high in this population. A recent employee survey reveals that employees would like more assistance to prevent and control these conditions, and they want opportunities at the work site to help them lose weight and quit smoking. This employer can use this information to cut costs, choose benefits, and provide opportunities for health promotion at the work site.

**3) Identify which clinical preventive services will address the risk profile of a population.** Employers have a variety of ways to determine which conditions, diseases, and risk factors are the most prevalent or costly to the company. They can get this information by conducting health risk appraisals, by reviewing data on medical or disability claims, by conducting medical evaluations of employees, or by doing a combination of these and using other sources. Employers can use these results to establish a group risk profile for their beneficiaries and then determine which services would be the most beneficial to address specific risks. An example of one scenario for an employer is described in the sample scenario in Box 1.

**4) Review the evidence statements before choosing clinical preventive services to offer as benefits.** Chapter 3 includes evidence statements for each of the recommended clinical preventive services. In the sample scenario, the employer wants to know more about the evidence for each recommended preventive service that will address those conditions and risk factors that are prevalent among its beneficiaries. Each evidence statement includes clinical recommendations made by USPSTF and other groups; condition- and disease-specific information; and information about the strength of the evidence, the economic value of the preventive intervention, and how the intervention should be delivered.

On the basis of the evidence statements, employer A decides to implement high-value clinical preventive services and benefits that address the conditions identified in the scenario. Employer A also decides to provide safe-harbor coverage for these preventive services in HSA-qualified plans and to eliminate co-pays for these preventive services in health maintenance organizations, preferred provider organizations, and point-of-service plans. The employer also works with a business coalition to establish low-cost interventions at the work site, such as tobacco-free policies; to provide access to low-cost, nutritious foods in the cafeteria and vending machines; and to place mileage markers on a walking course for employees.

**5) Identify recommended SPD language and Current Procedural Terminology (CPT) codes for the health plan contract.** After reviewing the evidence, employer A and its health benefits consultant decide to improve coverage of selected health benefits. They review chapter 2 to identify the SPD language statements for each of the recommended clinical preventive services they wish to include within their medical benefit plans. The employer plans to implement benefits to help beneficiaries lead healthy lifestyles to prevent and control the risk factors and conditions that lead to costly heart attacks and strokes. Employer A reviews the *Purchaser's Guide* and the American Heart Association and American College of Cardiology guidelines (21,22) and decides to cover the following SPD recommendations, on the basis of data about its employees and the needs of its beneficiaries:

- Aspirin therapy
- Screening for depression
- Screening for diabetes
- Counseling on a healthy diet
- Screening, counseling, and treatment for lipid disorders
- Screening, counseling, and treatment for hypertension
- Screening for obesity
- Screening, counseling, and treatment for tobacco use

**Box 2. Summary Plan Description Language: Hypertension**

**Hypertension (Screening)**

Covered screening includes conventional measurement with an arm cuff and an appropriately validated aneroid (containing no liquid) or digital sphygmomanometer (blood pressure meter).

Initiation, cessation, and interval of screening is a covered benefit for all children, adolescents, and adults and may be conducted as medically indicated.

**Hypertension (Counseling and Treatment) **

Covered treatment for hypertension includes

- Counseling to promote therapeutic lifestyle changes
- Office visits to monitor hypertension and treatment efforts
- Medications used to treat hypertension

Six counseling, treatment, and monitoring sessions are covered per calendar year. Additional counseling sessions are covered as medically indicated. Beneficiaries undergoing treatment with hypertension-lowering medications qualify for additional medication management visits, as medically indicated.

An example of the SPD for hypertension is presented in Box 2. CPT codes are provided in the *Purchaser's Guide* as an appendix to the SPD language statements to ensure that clinical preventive services are implemented and reimbursed. The American Medical Association developed the CPT codes, which are updated annually, to provide a uniform language that describes medical, surgical, and diagnostic services provided by clinicians. Employers who adopt the SPD recommendations in the *Purchaser's Guide* should ensure that administrators of their health plan approve the listed CPT codes for reimbursement of providers.

**6) Determine whether to change or remove coverage for services with limited or conflicting evidence of effectiveness or with potential adverse effects.** Some preventive services are very effective; others are ineffective, lack proof of effectiveness, or may be harmful. In chapter 5, the *Purchaser's Guide* summarizes the USPSTF ratings of clinical preventive services that were not included as recommendations using the following codes:

- I (insufficient evidence to recommend for or against)
- C (no recommendation either for or against)
- D (recommend against)

Employers should evaluate their current benefits and consider removing benefits for services that are ineffective, lack sufficient evidence, or are harmful.

**7)** **Look for opportunities to promote the delivery and use of preventive services. **Providing coverage for clinical preventive services is an essential step to improving overall employee health. Although coverage is necessary to promote the delivery and use of such services, coverage alone is not sufficient to optimize the health of employees. Employers need to ensure that health plan administrators, providers, and beneficiaries know that these benefits exist and should be used. Chapter 6, "Leveraging Benefits," identifies numerous ways that employers can promote the delivery and use of preventive services to influence healthy behavior, such as the following:

- Educate beneficiaries about the importance of clinical preventive services in adopting healthy lifestyles.
- Encourage beneficiaries to use covered preventive services appropriately.
- Support communitywide activities in disease prevention and health promotion.

**Box 3. Employer Success Story: Fieldale Farms**

Fieldale Farms, a poultry processor in Georgia, offered mobile screening, a gift card, and other incentives to all employees participating in its wellness program. Those with elevated blood pressure and high cholesterol were offered follow-up nutrition counseling on-site as a covered service and received company-paid fitness memberships. Over a 5-year period, 26% of the participating employees with high blood pressure and high cholesterol normalized these conditions through diet changes, medications, and physical activity. For 2004, the overall health care cost for an employee participating in the Fieldale Farms Wellness Program averaged $3052, less than half the national average health care cost for a manufacturing employee ($6900) (23).

State and local health departments and other organizations can assist employers in establishing connections with community-based resources to enhance use of preventive services. Chapter 6 also includes information about the CDC *Community Guide*, which directs health care purchasers to evidence-based recommendations for population health that may enhance their investment in clinical preventive services. The *Community Guide *also gives employers examples of activities to improve the delivery and use of preventive services, such as using client reminders, incentives, media, and feedback to providers. This guide is available at www.thecommunityguide.org. This chapter also gives examples of employers' successes (Box 3).

**8) Establish and evaluate goals for the health of beneficiaries.** Employers should consider establishing goals and benchmarks to evaluate whether employees'  health has improved over time. Chapter 7, "Resources and Tools," provides life-course charts that provide a visual guide to clinical preventive services across the life span. The chapter also charts and compares the recommendations proposed in the *Purchaser's Guide *with the 2007 Healthcare Effectiveness Data and Information Set (HEDIS), the National Committee for Quality Assurance's *2007* *State of Health Care Quality Report*, and the U.S. Department of Health and Human Services' *Healthy People 2010: With Understanding and Improving Health and Objectives for Improving Health (HP 2010).* In the sample scenario, for example, employer A can decide to use any of the goals listed in* HP 2010 *or the 2007 HEDIS measures as benchmarks for its beneficiaries. For example, the *HP 2010* target is to reduce the proportion of adults aged 20 years or older with hypertension to 16% and to increase to 50% the proportion of adults aged 18 years or older who have their high blood pressure under control. Employer A could track progress toward these goals.

## Discussion

*A Purchaser's Guide to Clinical Preventive Services: Moving Science into Coverage* is an important tool for employers. The *Purchaser's Guide* translates clinical guidelines and medical evidence into lay terms, providing employers with the information they need to select, define, and implement 46 preventive medical benefits. Employers and health benefits consultants can use the *Purchaser's Guide* to develop a benefits package that includes preventive services proven to be effective in preventing illness and premature death.

The *Purchaser's Guide* has some limitations. Although the clinical recommendations are based on strong evidence, information pertaining to the economic benefits was lacking for some recommendations. Even so, each chapter represents the best available evidence in the published literature. Second, although the *Purchaser's Guide* includes advice for improving coverage, it does not comprehensively address the policy, educational, and system interventions necessary to ensure that clinicians deliver the preventive services and that patients can access them. Third, the comprehensiveness of the recommendations could be overwhelming to employers in a resource-constrained environment. On the other hand, the *Purchaser's Guide* provides different scenarios to help employers prioritize and select the services that may be most useful to their beneficiaries.

Clinical preventive services can reduce health care costs by preventing disease, injury, and disability and by identifying health problems early, when treatment is most effective and least expensive. Employers play a powerful role in shaping health care for many people in the United States. They decide what preventive health care services to cover in an employee's health plan and whether to establish work site programs and policies in health promotion that support healthy lifestyles.

The *Purchaser's Guide* promotes the application of consistent standards for quality care, which will provide beneficiaries better value in their health plan. Perhaps most importantly, the *Purchaser's Guide* provides succinct guidance to help employers develop an overall strategy to phase in effective preventive benefits and to customize preventive programs for their employees. NBGH reports that many Fortune 500 companies will be using the *Purchaser's Guide* when negotiating their health benefit contracts and developing their health care strategies, which could improve the health of millions of employees and their dependents.

In an era of limited resources and increased need for accountability to improve health outcomes, the *Purchaser's Guide* helps employers design value-based quality health plans. As employers address rising health care costs and the growing burden of chronic disease in the United States, the *Purchaser's Guide* will be a useful tool for developing and evaluating health benefits plans, and will allow employers to improve the preventive health services they offer.

## Figures and Tables

**Table 1:** *A Purchaser's Guide to Clinical Preventive Services:* Description of Chapters

Chapter	Title	Description
1	The Role of Clinical Preventive Services in Disease Prevention and Early Detection	Information for employers on improving the health of beneficiaries and reducing health care costs by implementing a comprehensive and structured benefit for clinical preventive services within a medical benefit plan.
2	Summary Plan Description (SPD) Language Statements for Recommended Clinical Preventive Services Benefits	Forty-six condition-specific SPD language statements designed to assist benefits staff as they design, discuss, negotiate, and set benefit structures and coverage guidelines with a health plan, union, or consumer group.
3	Evidence Statements for Recommended Clinical Preventive Service Benefits	Scientific evidence behind each of the 72 recommendations for benefits (i.e., screening, testing, counseling, immunization, preventive medication, and preventive treatment).
4	The Prioritization and Strategic Implementation of Clinical Preventive Service Benefits	Practical advice and employer scenarios regarding the strategic implementation of benefits.
5	Clinical Preventive Services: Recommendations and Statements of the U.S. Preventive Services Task Force (USPSTF)	Information on clinical preventive services that were reviewed by the USPSTF but not included in the *Purchaser's Guide.* This information may assist benefits staff in determining which clinical preventive services currently offered in the health plan(s) should be re-evaluated.
6	Leveraging Benefits: Opportunities to Promote the Delivery and Use of Preventive Services	Actions that employers can take to strengthen prevention efforts by supporting or implementing public health interventions in the workplace and in communities.
7	Resources and Tools	Additional information for employers on clinical preventive services, including "at a glance" guides to disease prevention throughout the life span and other helpful tools.

PDF copies of the *Purchaser's Guide* are available on the Web sites of the National Business Group on Health (www.businessgrouphealth.org/prevention/purchasers) and the Centers for Disease Control and Prevention  (www.cdc.gov/business).

**Table 2:** High-Value Preventive Services^a^(5), by Rank Order

Service	CPB —Maximum Score = 5	CE —Maximum Score = 5	Combined Score CPB and CE —Maximum Score = 10
Aspirin chemoprophylaxis	5	5	10
Childhood immunization series	5	5	10
Tobacco use, screening and brief intervention	5	5	10
Problem drinking, screening and brief counseling	4	4	8
Colorectal cancer screening	4	4	8
Hypertension screening	5	3	8
Influenza immunization	4	4	8
Pneumococcal immunization	3	5	8
Vision screening (adults)	3	5	8
Cervical cancer screening	4	3	7
Cholesterol screening	5	2	7
Breast cancer screening	4	2	6
Calcium chemoprophylaxis	3	3	6
Chlamydia screening	2	4	6
Vision screening (children)	2	4	6
Folic acid chemoprophylaxis	2	3	5
Obesity screening	3	2	5
Depression screening	3	1	4
Hearing screening	2	2	4
Injury prevention, counseling	1	3	4

CPB indicates clinically preventable burden; CE, cost-effectiveness.

a Evaluated in terms of the clinically preventable burden of disease and cost-effectiveness.
